# Should All Individuals Be Screened for Genetic Predisposition to Cancer?

**DOI:** 10.1155/2021/6611963

**Published:** 2021-01-09

**Authors:** Sarah Wedderburn, Terri P. McVeigh

**Affiliations:** ^1^West of Scotland Regional Genetics Service, Laboratory Medicine Building, Queen Elizabeth University Hospital, Glasgow, UK; ^2^Cancer Genetics Unit, Royal Marsden NHS Foundation Trust, London, UK

## 1. Background

The principles for screening were defined in 1968 by Wilson and Junger, with a central tenant—“*on the one hand, bringing to treatment those with previously undetected disease, and, on the other, avoiding harm to those persons not in need of treatment*” [1].

The question of whether genomic screening should be introduced to detect predisposition to cancer is complex. Its key elements are fourfold. “*Should*” implies a duty to screen. The duty would be if the benefit of screening outweighed harm and the potential benefits and harms need to be considered with respect to the patient, the wider population, and the economy. “*All individuals*” suggests indiscriminate application of screening test as opposed to a target population. Does “*Genetic Predisposition*” include monogenic changes, polygenic changes, or both? What about penetrance? Finally, “*Cancer*” needs further definition. All cancers? Solid tumours only? Particular types of cancer? To screen everyone for all genetic changes which can predispose to all types of cancer is ambitious to say the least. It would surpass the scale of any national screening programme to date. In order to answer this question, it is perhaps best to first consider the concept of a health-screening programme.

## 2. Genetic Variation

Thanks to evolution, human beings are genetically diverse. The estimated number of variants in the normal human genome is approximately 5,000,000, of which approximately 10,000 are protein changing, 150 are protein truncating, and an estimated 40–100 arise as de novo events [2]. The majority of these variants have only a slight, or no, deleterious impact on health. Most known disease-causing variants are located in the coding part of our DNA, which accounts for only 2% of our genomic material. Genetic testing using multigene panels or whole exome sequencing focuses on the coding sequence, but more recently, application of whole genome sequencing to detect cancer predisposing variation has come to the fore. Most genetic predisposition to cancer relates to sequence variation, but there are other genetic mechanisms which may influence cancer risk, such as methylation defects (constitutional *MLH1* hypermethylation [3]), imprinting disorders (e.g., Beckwith–Wiedemann syndrome [4]), and copy-neutral structural rearrangements [5] (e.g., *MSH2* inversion [6] and chromosome 3 translocations involving *VHL* [7]), which are not readily detected by whole genome sequencing.

### 2.1. Genetic Predisposition to Disease

Mendelian disorders are generally associated with variants in a single gene or family of genes, which are typically identified through linkage studies involving families affected with a specific disorder. Variants associated with common complex disorders are typically identified through large-scale genome-wide association involving cases compared to unaffected controls. Most such variants are intergenic rather than in the coding DNA and individually exert only a slight influence on risk. A polygenic risk score is the quantification of the cumulative effect of a number of genetic variants which individually have a small effect on susceptibility to disease of interest. Variants associated with cancer are associated with different risks, depending on patient age/gender, the type of variant, the gene/part of the gene in which they are located, and other coinherited genetic modifiers. The risk of cancer may also be modified further by lifestyle/environmental factors.

## 3. Genetic Screening for Cancer Predisposition

The World Health Organization defines screening as “the presumptive identification of unrecognised disease in an apparently healthy, asymptomatic population by means of tests, examinations or other procedures that can be applied rapidly and easily to the target population” [8]. Breast, bowel, and cervical cancers are the UK's main cancer screening programmes. These programmes have been guided by the screening criteria developed by Wilson and Junger in 1968.

The criteria can be split into three main components based on the questions set by Wilson and Junger [9] (Table 1 in Supplementary Materials): the disease, screening test, and diagnosis and treatment.

### 3.1. The Disease

There is no doubt that cancer is an important health problem. The principle cause of death globally is cardiovascular disease, and cancer is second [10]. The depth of our knowledge and understanding surrounding the natural history of cancer is constantly improving. Many cancers have well-defined natural history, such as the adenoma-carcinoma sequence of colorectal cancer and carcinoma in situ to invasive breast cancer, while others are less clear cut (p53 signature, serous tubal intraepithelial cancer to ovarian cancer, etc.). Those cancers associated with cancer predisposition syndromes may be associated with alternative or accelerated natural histories (e.g., mismatch repair crypts—carcinoma in patients with Lynch syndrome). The time lag from an asymptomatic to symptomatic patient is highly variable and dependent on a multitude of factors. Genetically, predisposed carriers of variants associated with incomplete penetrance may never develop a cancer but have a higher risk of doing so and generally at younger ages than the same cancers in the general population. However, the time lag is usually sufficient to enrol an individual into a relevant screening/surveillance programme or to initiate surgical/chemoprophylaxis, where appropriate risk-reduction strategies are available.

### 3.2. The Screening Test

The ACCE model proposed by the Centers for Disease Control and Prevention, Office of Public Health Genomics suggests that a genetic test should be evaluated based on four key criteria, namely, analytical validity, clinical validity, clinical utility, and ethical, legal, and societal implications [11], metrics that have been further expanded by the UK Genetic Testing Network to include evidence of gene-disease association, and feasibility of test delivery [12].

Assessment of analytical validity should include consideration of analytical sensitivity and specificity as well as clinical sensitivity and specificity (Table 2 in Supplementary Materials) [13, 14]. Assessment of clinical sensitivity/specificity is particularly challenging when considering tests in patients with cancer, considering the broad genetic heterogeneity and possible contribution of polygenic risk. Assessment of such parameters is also challenging in the case of patients without a cancer phenotype.

Most contemporary genetic testing is performed using next-generation technology but can vary from single gene to many dozens or even hundreds of genes of interest. More recently, whole exome sequencing (WES) and whole genome sequencing (WGS) tests have become available in the diagnostic setting. Analysis of data arising from WES/WGS may be focused to specific genes/regions of interest, so-called “virtual panels,” or may be unrestricted, with the potential of inadvertently identifying incidental findings (Table 3 in Supplementary Materials). As genomic testing becomes increasingly cost-effective, multigene panels are becoming bigger, incorporating numerous genes, many of which have a weak or unproven association with cancer risk. As the number of genes included on panels increases, so too does the likelihood of identifying a pathogenic variant, as does the likelihood of identifying variants of uncertain significance [15].

All tests have limitations, and it is important to take into account that certain regions of the human genome are technically challenging to sequence, such as repetitive or, GC-rich regions, and may be complicated by the presence of pseudogenes [13]. Cautious interpretation of negative results related to regions of the genome where coverage is suboptimal is recommended where clinical suspicion is high.

The practical issues around delivery of germline genetic tests include shortages in expertise to counsel and consent patients appropriately and staff to accurately perform the test and analyse the results.

Standards and guidance to genetic variant interpretation was published by the ACMG in 2017 [16]. The aim was to provide consensus on how laboratories and clinicians across the world interpret genetic results. In the UK, the Cancer Variant Interpretation group provides a national forum for variant interpretation to help standardise practices in laboratory hubs [17]. Other international expert panels (ClinGen expert groups [18], ENIGMA [19], and INSiGHT [20]) represent a global multidisciplinary effort to guide variant classification. Despite availability of global expertise, variant interpretation and classification is complex, particularly if a variant is identified in a patient without a readily available phenotype. Many variants cannot be definitively classified as pathogenic or benign, thus being classified as “variants of uncertain significance.” Benign variants are rarely reported back to the patient. Communication of a pathogenic result is relatively straightforward (depending on gene and estimated associated risks). Explaining that a variant of uncertain significance (VUS) has been identified is less so. There is a lack of global consensus about how variants of uncertain significance should be followed (how regularly or by whom). Some laboratories/clinical services maintain registers, perform systematic reinterpretation regularly; other services lack the workforce capacity to provide any such systematic review [21]. Patient ability to deal with an uncertain result, like a VUS, has been shown to hinge on their pretest and posttest counselling: the adequacy and clarity of explaining a VUS is key [22]. Patient anxiety and distress is something which should be addressed as potential harm. Female carriers of *BRCA1/2* VUS have been shown to have greater levels of anxiety and distress when compared to females with definite pathogenic or benign results [23]. However, when a result is pathogenic, the potential benefits are only as good as the onward screening which can be offered.

Whilst the costs of genetic tests are falling, as illustrated by the cost of sequencing a whole genome following the prediction of Moore's law [24], this is not the whole economic picture. Affordability of cancer predisposition screening should consider all relevant costs. This should encompass the patient journey from screening invite, delivery of results, to any necessary screening or treatment. Multiple highly skilled staff members are required to facilitate genetic screening. Raw material costs from blood tubes and primers and even the cost of postage should be included. There would also be the initial outlay cost to set up such a screening programme. Thereafter, the associated costs of cancer diagnosis and treatment are cancer type-dependent.

## 4. Risk Estimation and Mitigation

Well-established cancer screening programmes show that early intervention can improve clinical outcomes. In patients with hereditary cancer predisposition syndromes, mortality rates can be significantly mitigated by screening, such as colonoscopy in Lynch syndrome or MRI/mammograms in hereditary breast and ovarian cancer syndrome [25, 26]. For certain types of cancers, or for genes associated with broad poorly defined phenotype, a robust screening test may not be readily available, either because a screening test has not been proven to confer survival advantage or because the public health system cannot readily provide access to same [27, 28]. For a number of cancers, prophylactic surgery may not be feasible, and for others, the potential risk associated with prophylactic surgery may outweigh the potential benefit. Where an onward screening exists, there is a potential benefit for the patient and associated cost-effectiveness for the wider economy, the aim being to detect disease early and therefore at a more treatable/curable stage. If there is no onward screening, the patient may be informed that they are at increased risk, without a clear management plan. This can cause anxiety, distress, uncertainty, and ultimately harm for the patient.

Once pathogenicity of a variant has been established, questions still remain with respect to penetrance and expressivity. The cancer risk associated with a pathogenic variant is gene specific and may be further modified by other environmental and genomic factors. Cancer risk estimates for pathogenic variants in particular genes and recommendations for management of carriers are broadly based on epidemiological studies of highly selected groups. Traditional criteria for genetic testing aim to identify patients with a high *a priori* probability of variant detection based on their personal and family history. The risk estimates for patients with a high-risk personal and family history of cancer may be vastly in excess of those in unselected patients detected as part of a population genetic screening initiative. This has significant implications in feasibility and cost-effectiveness of associated cancer surveillance, with increasing numbers needed to screen to detect one cancer. Furthermore, this has implications for surgical and anaesthetic workload, as many carriers may choose risk-reducing surgery. There is some evidence to suggest that population-level screening for highly penetrant genetic variants predisposing to potentially preventable cancers may be cost-effective [29].

## 5. Discussion

Having considered the screening criteria developed by Wilson and Junger, it is clear that there is some way to go before all individuals could be screened for genetic predisposition to cancer. A different question to ask that may be more readily feasible is “Could we screen all individuals for predisposition to certain cancers?”

According to UK cancer statistics, the most common cancers are breast, bowel, lung, and prostate [30]. These account for >50% of new cancer cases per year. Breast cancer is the most common cancer in women and prostate the most common in men [31]. The commonest heritable cancers are breast and bowel cancer. There are already national clinical screening programmes in place for breast and bowel cancers, as well as programmes tailored to hereditary forms of these cancers. Therefore, screening for genetic predisposition to breast and bowel cancers would be logical areas to explore. Ovarian cancer, though less common, is associated with high cost of treatment and high mortality but has a significant heritable contribution and may be prevented if early risk-reducing bilateral salpingo-oophorectomy is undertaken. Population-based genetic screening for high-risk variants predisposing to this type of cancer has already been shown to be cost-effective [29].

An important consideration is whether or not individuals actually want genetic screening. There is evidence that the general population have an increased awareness of genetic testing and cancer risk leading to an inevitable increase in demand [32]. The advent of direct-to-consumer testing has already enabled individuals to explore their genetic risk independently with the inevitable result that clinicians become involved when the result is reported as anything other than “normal” [33]. Overall, the population has positive attitudes towards genetic testing [34]. The best known example of increased uptake in genetic testing is for breast and ovarian cancers and can be traced back to the “Angelina Jolie effect” [35]. Data from the UK female population highlight that they are already accepting population breast and ovarian cancer genetic testing with consistency across different demographics [36]. These factors are encouraging and generally support a willingness from the population for population genetic screening.

The ethnicity of the people in the screened cohort should be considered in determining feasibility of a genetic screening programme and numbers needed to test to detect a mutation carrier. Individuals of Ashkenazi Jewish descent, for example, have a prior probability of carrying a pathogenic variant in *BRCA1* or *BRCA2* of 1 in 40, compared to a frequency of 1/195 in non-Finnish Europeans [37]. Individuals of non-European ancestry are largely underrepresented in population-level genomic studies, and so carrier frequency in this patient population is less well defined. This fact also increases challenges in variant interpretation.

Adequate genetic counselling would be critical if offering a population screening programme;it has been purported that genetic testing without appropriate pretest counselling may be more risky than beneficial. Current literature focuses on individuals undergoing *BRCA* testing. Importantly, there is currently no data from a randomised control trial which has considered pretest counselling vs no pretest counselling in population *BRCA* testing [38]. But, when considering the psychological impact of *BRCA* testing, there is much conflicting evidence [39]. The common themes are that cancer-related distress and anxiety are significantly increased in those who carry a pathogenic variant, particularly in the short term, with levels generally returning to normal with time [40, 41]. For individuals who receive a VUS, there would be an increased need for support and counselling to understand the implications of the result and what prophylactic/screening options are available to the individual [42]. Younger women in particular have been highlighted as a more vulnerable group in *BRCA1* testing [43]. This can be extrapolated to other young individuals undergoing cancer genetic testing. For example, such individuals may be too young to enter a particular screening programme, or they may feel constraint around life choices, like having children at a younger age than they originally planned [38]. In contrast to these concerns, a recent study investigating population *BRCA* testing in the North London Ashkenazi Jewish population was very positive about population screening. It found that an economically suitable programme could be delivered which included adequate pretest counselling [33]. More widespread delivery of genetic counselling at population scale will be a significant challenge. The resources required to counsel individuals both before and after a test as necessary are vast. The clinical genetics services currently do not have the capacity for this [44]. Digital media could provide an alternative or adjunct to face-to-face counselling [45]; an interactive online hub for the target screening population could be a solution. This would require considerable initial investment when setting up a screening programme.

Genetic laboratories across the UK are similarly under pressure to address current demands for diagnostic testing. The laboratory hubs currently offer a variety of cancer gene tests, which are currently easily and rapidly accessible for patients meeting testing criteria within the NHS. The volume of genetic test requests would significantly increase if a screening programme was implemented, and the current service offered by genetic laboratories would not be sustainable. Again, significant investment in laboratories, bioinformatics support, and secure data solutions would be required.

The interpretation of genetic variants is another potential pitfall. Variants can be upgraded or downgraded as and when evidence changes. A system to account for this would need to be established. There are multiple variant reclassification scenarios, all of which can have implications for the individual(s) and families whose result it is. The possibility of results changing would have to be addressed when individuals consent to genetic testing.

The recent update on Consent and Confidentiality in Genomic Medicine provides a Record of Discussion regarding testing and/or storage of genetic material [46]. This can be used for mainstream genetic testing and so in theory, it could be a suitable basis for consent to genetic screening tests. The issue of insurance would also need to be addressed before an individual undertook genetic testing. Identifying a pathogenic variant can have significant implications in this field.

Issues discussed so far primarily address issues around monogenic germline variants. However, it is more complicated in reality. Polygenic risk has not been addressed. Polygenic risk is a culmination of common, rare, and intermediate genetic variants and their interactions which influence an individual's susceptibility to disease [47]. Polygenic risk plays a greater overall role in cancer susceptibility than monogenic variants [48], but the practicalities of assessing this pose significant hurdles computationally [23].

Several hundred low-risk alleles that influence cancer risk have been identified. Polygenic risk scores (PRS) attempt to quantify the cumulative effect of a number of common genetic variants which individually have a small effect on susceptibility to the disease of interest. The polygenic risk score can only be used to provide information about risk relative to the population in which it was derived—it is not possible to generate an absolute risk estimate. PRS follow a normal distribution across a population, such that individuals in the 90% centile have a risk of disease that is several-fold higher than those individuals in the 10^th^ centile. PRS can be significantly influenced by ethnicity, with many PRS based on European White populations. PRS must also be interpreted with consideration of the patient's age. PRS to predict future cancer risk in unaffected individuals are being investigated on a research basis in a number of studies, including the BARCODE1 study, which aims to determine the utility of a custom-designed PRS in stratifying the risk of prostate cancer in men in the general population to inform targeted screening. For a comprehensive assessment of an individual's heritable cancer risk, testing to include monogenic and polygenic risk factors should be undertaken, and the interpretation of such risk scores should be carefully considered in the context of the wider family history and other heritable mechanisms of disease that may not be detected using standard next-generation techniques. The use of PRS in stratifying cancer risk in carriers of high-risk variants in *BRCA1*, *BRCA2*, or other genes, may soon become available for clinical use [49–51].

Modifiable risk factors also have their part to play. These account for approximately 35% of cancer deaths globally [52]. Should factors such as smoking, obesity, and alcohol consumption be included towards an individual's risk? It could be argued that focus should be on tackling modifiable risk factors as opposed to nonmodifiable risk factors. This could give a greater net benefit to the population as a whole.

There are many more points which could be discussed, all at considerable length, but they are beyond the scope of this discussion. Given the complexity of genetic predisposition to cancer for each individual, implementing an appropriate screening programme for this is simply not feasible at present. *Should* is arguably the key word in the question posed. One thing this discussion has not addressed is the ethics of such screening. However, when only considering the current state of the NHS and the pressures it faces on its limited resources, I would argue that we should not screen all individuals for genetic predisposition to cancer. Screening programmes need considerable health resources, robust infrastructure, and capacity within the country's health care system to cope. To screen all individuals for genetic predisposition to cancer in the broadest sense would make demands which the NHS and current genetics services simply could not fulfil. A more refined screening programme could be explored and lessons learned could guide any future programmes.

*“In theory, screening is an admirable method of combating disease … [but] in practice, there are snags.”* [1]

## Supplementary Materials

Supplementary Materials Table 1: the key elements of Wilson and Junger screening criteria, 1968 [9]. Table 2: definitions for clinical and analytical sensitivity and specificity [8]. Table 3: a comparison of contemporary genetic testing. ^∗^ indicates gene-dependent, which can be more expensive than gene panels.

## References

[1] Wilson J. M. G., Jungner G. (1968). *Principles and Practice of Screening for Disease*.

[2] Auton A., Auton A., Brooks L. D. (2015). A global reference for human genetic variation. *Nature*.

[3] Pinto D., Pinto C., Guerra J. (2018). Contribution of MLH1 constitutional methylation for Lynch syndrome diagnosis in patients with tumor MLH1 downregulation. *Cancer Medicine*.

[4] Mussa A., Molinatto C., Baldassarre G. (2016). Cancer risk in Beckwith-Wiedemann syndrome: a systematic review and meta-analysis outlining a novel (Epi)Genotype specific histotype targeted screening protocol. *The Journal of Pediatrics*.

[5] Morak M., Steinke-Lange V., Massdorf T. (2020). Prevalence of CNV-neutral structural genomic rearrangements in MLH1, MSH2, and PMS2 not detectable in routine NGS diagnostics. *Familial Cancer*.

[6] Mork M. E., Rodriguez A., Taggart M. W. (2017). Identification of MSH2 inversion of exons 1-7 in clinical evaluation of families with suspected Lynch syndrome. *Familial Cancer*.

[7] Van Erp F., Van Ravenswaaij C., Bodmer D. (2003). Chromosome 3 translocations and the risk to develop renal cell cancer: a Dutch intergroup study. *Genetic Counseling*.

[8] Health Knowledge, 2019, https://www.healthknowledge.org.uk/public-health-textbook/disease-causation-diagnostic/2c-diagnosis-screening/principles-methods-applications

[9] Andermann A., Blancquaert I., Beauchamp S., Déry V. (2008). Revisting Wilson and Jungner in the genomic age: a review of screening criteria over the past 40 years. *Bulletin of the World Health Organization*.

[10] Krstic M. N., Mijac D. D., Popovic D. D., Markovic A. P., Milosavljević T. (2019). General aspects of primary cancer prevention. *Digestive Diseases*.

[11] https://www.cdc.gov/genomics/gtesting/acce/

[12] Burke W., Zimmern R. (2007). Moving beyond ACCE: an expanded framework for genetic test evaluation pdf icon [344 kB] external icon. *A Paper for the United Kingdom Genetic Testing Registry, *.

[13] Association for Clinical Genomic Science (2015). *Practice Guidelines for Targeted Next Generation Sequencing Analysis and Interpretation*.

[14] Burke W. (2014). Genetic tests: clinical validity and clinical utility. *Current Protocols in Human Genetics*.

[15] Tsaousis G. N., Papadopoulou E., Apessos A. (2019). Analysis of hereditary cancer syndromes by using a panel of genes: novel and multiple pathogenic mutations. *BMC Cancer*.

[16] Richards S., Aziz N., Aziz N. (2015). Standards and guidelines for the interpretation of sequence variants: a joint consensus recommendation of the American College of Medical Genetics and Genomics and the Association for Molecular Pathology. *Genetics in Medicine*.

[17] Garrett A., Callaway A., Durkie M. (2020). Cancer variant interpretation group UK (CanVIG-UK): an exemplar national subspecialty multidisciplinary network. *Journal of Medical Genetics*.

[18] https://clinicalgenome.org/affiliation/

[19] https://enigmaconsortium.org/

[20] https://www.insight-group.org/about/

[21] McVeigh T. P., Kelly L. J., Whitmore E. (2019). Managing uncertainty in inherited cardiac pathologies—an international multidisciplinary survey. *European Journal of Human Genetics*.

[22] Skinner D., Roche M. I., Weck K. E. (2018). “Possibly positive or certainly uncertain?”: participants’ responses to uncertain diagnostic results from exome sequencing. *Genetics in Medicine*.

[23] Murray M. L., Cerrato F., Bennett R. L., Jarvik G. P. (2011). Follow-up of carriers of BRCA1 and BRCA2 variants of unknown significance: variant reclassification and surgical decisions. *Genetics in Medicine*.

[24] Hayden E. C. (2014). Technology: the $1,000 genome. *Nature*.

[25] Møller P., Seppälä T., Bernstein I. (2017). Cancer incidence and survival in Lynch syndrome patients receiving colonoscopic and gynaecological surveillance: first report from the prospective Lynch syndrome database. *Gut*.

[26] Kurian A. W., Sigal B. M., Plevritis S. K. Survival analysis of cancer risk reduction strategies for BRCA1_2 mutation carriers. *Journal of cliNical Oncology*.

[27] The Lancet Gastroenterology Hepatology (2019). Pancreatic cancer screening: more harms than benefits?. *Lancet Gastroenterology Hepatology*.

[28] Mathieu K. B., Bedi D. G., Thrower S. L., Qayyum A., Bast R. C. (2018). Screening for ovarian cancer: imaging challenges and opportunities for improvement. *Ultrasound in Obstetrics & Gynecology*.

[29] Manchanda R., Patel S., Gordeev V. S. (2018). Cost-effectiveness of population-based BRCA1, BRCA2, RAD51C, RAD51D, BRIP1, PALB2 mutation testing in unselected general population women. *JNCI: Journal of the National Cancer Institute*.

[30] https://www.cancerresearchuk.org/health-professional/cancer-statistics/incidence

[31] Cancer Research UK, 2019, https://www.cancerresearchuk.org/health-professional/cancer-statistics/incidence/common-cancers-compared

[32] Laforest F., Kirkegaard P., Mann B., Edwards A. (2019). Genetic cancer risk assessment in general practice: systematic review of tools available, clinician attitudes, and patient outcomes. *British Journal of General Practice*.

[33] Peterson E. B., Chou W.-Y. S., Gaysynsky A. (2018). Communication of cancer-related genetic and genomic information: a landscape analysis of reviews. *Translational Behavioral Medicine*.

[34] Hann K. E. J., Freeman M., Fraser L. (2017). PROMISE study team. Awareness, knowledge, perceptions, and attitudes towards genetic testing for cancer risk among ethnic minority groups: a systematic review. *BMC Public Health*.

[35] McCuaig J., Armel S., Care M., Volenik A., Kim R., Metcalfe K. (2018). Next-generation service delivery: a scoping review of patient outcomes associated with alternative models of genetic counseling and genetic testing for hereditary cancer. *Cancers*.

[36] Meisel S. F., Rahman B., Side L. (2016). PROMISE-2016 study team. Genetic testing and personalized ovarian cancer screening: a survey of public attitudes. *BMC Womens Health*.

[37] Maxwell K. N., Domchek S. M., Nathanson K. L., Robson M. E. (2016). Population frequency of germline BRCA1/2 mutations. *Journal of Clinical Oncology*.

[38] Manchanda R., Burnell M., Gaba F. (2020). Randomised trial of population‐based BRCA testing in Ashkenazi Jews: long‐term outcomes. *BJOG: An International Journal of Obstetrics & Gynaecology*.

[39] Lombardi L., Bramanti S. M., Babore A. (2019). Psychological aspects, risk and protective factors related to BRCA genetic testing: a review of the literature. *Supportive Care in Cancer*.

[40] Lumish H. S., Steinfeld H., Koval C. (2017). Impact of panel gene testing for hereditary breast and ovarian cancer on patients. *Journal of Genetic Counseling*.

[41] Oliveri S., Ferrari F., Manfrinati A., Pravettoni G. (2018). A systematic review of the psychological implications of genetic testing: a comparative analysis among cardiovascular, neurodegenerative and cancer diseases. *Frontiers in Genetics*.

[42] Jacobs C., Patch C., Michie S. (2019). Communication about genetic testing with breast and ovarian cancer patients: a scoping review. *European Journal of Human Genetics*.

[43] Brunstrom K., Murray A., McAllister M. (2016). Experiences of women who underwent predictive BRCA1/2 mutation testing before the age of 30. *Journal of Genetic Counseling*.

[44] Hoskovec J. M., Bennett R. L., Carey M. E. (2018). Projecting the supply and demand for certified genetic counselors: a workforce study. *Journal of Genetic Counseling*.

[45] Metcalfe S. A. (2018). Genetic counselling, patient education, and informed decision-making in the genomic era. *Seminars in Fetal and Neonatal Medicine*.

[46] Royal College of Physicians (2019). *Consent and Confidentiality in Genomic Medicine: Guidance on the Use of Genetic and Genomic Information in the Clinic*.

[47] Chatterjee N., Shi J., García-Closas M. (2016). Developing and evaluating polygenic risk prediction models for stratified disease prevention. *Nature Reviews Genetics*.

[48] Khera A. V., Chaffin M., Aragam K. G. (2018). Genome-wide polygenic scores for common diseases identify individuals with risk equivalent to monogenic mutations. *Nature Genetics*.

[49] Eeles R. A., Raghallaigh H. N. (2020). BARCODE 1: a pilot study investigating the use of genetic profiling to identify men in the general population with the highest risk of prostate cancer to invite for targeted screening. *Journal of Clinical Oncology*.

[50] Barnes D. R., Rookus M. A., McGuffog L. (2020). Polygenic risk scores and breast and epithelial ovarian cancer risks for carriers of *BRCA1* and *BRCA2* pathogenic variants. *Genetics in Medicine: Official Journal of the American College of Medical Genetics*.

[51] Gallagher S., Hughes E., Wagner S. (2020). Association of a polygenic risk score with breast cancer among women carriers of high- and moderate-risk breast cancer genes. *JAMA Network Open*.

[52] Lewandowska A., Rudzki M., Rudzki S., Lewandowski T., Laskowska B. (2019). Environmental risk factors for cancer—review paper. *Annals of Agricultural and Environmental Medicine*.

