# Supplementary material for: Should All Individuals Be Screened for Genetic Predisposition to Cancer?
Source: Genet Res (Camb). 2021 Jan 9;2021:6611963. doi: 10.1155/2021/6611963 (PMC7953527; doi:10.1155/2021/6611963)
Supplement: Supplementary Materials — Table 1: the key elements of Wilson and Junger screening criteria, 1968 [9]. Table 2: definitions for clinical and analytical sensitivity and specificity [8]. Table 3: a comparison of contemporary genetic testing. ∗ indicates gene-dependent, which can be more expensive than gene panels. [file 6611963.f1.docx]

Table 1: The key elements of Wilson and Junger screening criteria, 1968^9^

| Component | Question |
| --- | --- |
| The Disease | 1. Is it an important health problem? |
|  | 1. 2 Is the natural history well understood? |
|  | 1. Is there a long time between the presence of risk factors/sub-clinical disease to overt disease? |
|  | 1. Does early intervention improve clinical/public health outcome? |
| Screening Test | 1. Is the test valid (sensitivity and specificity)? |
|  | 1. Is the test simple, reliable and affordable? |
|  | 1. Is the test acceptable to patient and staff? |
| Diagnosis and Treatment | 1. Is access to diagnostic facilities available and rapid? |
|  | 1. Is treatment effective and accessible? |
|  | 1. Is it cost-effective? |
|  | 1. Is it sustainable? |
|  | 1. Does benefit outweigh the harm? |

| Metric | Definition |
| --- | --- |
| Analytical Sensitivity | The proportion of DNA samples with a positive result/known variant which are correctly classed as positive |
| Analytical Specificity | The proportion of DNA samples with a negative result/no known variant which are correctly classed as negative |
| Clinical Sensitivity | The proportion of individuals, with a specific condition, who have a test result which is positive |
| Clinical Specificity | The proportion of individuals, without a specific condition, who have a test result which is negative |

Table 2. Definitions for Clinical and Analytical Sensitivity and Specificity^8^

| Type of sequencing | Single Gene | Gene Panel | Clinical Exome | Whole Genome |
| --- | --- | --- | --- | --- |
| Scope of Test | Disease-causing gene | Disease-causing and associated genes | 2% of genome, all clinically known genes | All coding and non-coding DNA |
| Variants produced | Minimal |  |  | -4,000,000 |
| Incidental Findings | None |  |  | Potential to find |
| Cost | Least expensive^*^ |  | | Most expensive |

Table 3. *gene dependent, can be more expensive than gene panels
